# Psychometric properties of the Korean version of the medical outcomes study HIV health survey: results from a multicenter survey in Korea

**DOI:** 10.1186/s12955-018-0919-6

**Published:** 2018-05-15

**Authors:** Eun-Jung Shim, Hyeju Ha, Sun Hee Lee, Nam Joong Kim, Eu Suk Kim, Ji Hwan Bang, Kyoung-Ho Song, Bo Kyung Sohn, Hye Youn Park, Kyung-Lak Son, Heesung Hwang, Kwang-Min Lee, Bong-Jin Hahm

**Affiliations:** 1 Department of Psychology, Pusan National University, Busan, Republic of Korea; 2 Department of Internal Medicine, Pusan National University School of Medicine, Busan, Republic of Korea; 3 Department of Internal Medicine, Seoul National University College of Medicine, Seoul, Republic of Korea; 4 Department of Internal Medicine, Seoul National University Bundang Hospital, Seongnam, Republic of Korea; 5 Division of Infectious Diseases, Seoul National University Seoul Metropolitan Government Boramae Medical Center, Seoul, Republic of Korea; 6 Department of Psychiatry, Sanggye Paik Hospital, Seoul, South Korea; 7 Department of Psychiatry, Inje University College of Medicine, Busan, Republic of Korea; 8 Department of Neuropsychiatry, Seoul National University Bundang Hospital, Seongnam, Republic of Korea; 9 Department of Neuropsychiatry, Seoul National University Hospital, Seoul, Republic of Korea; 10 Department of Psychiatry and Behavioral Sciences, Seoul National University College of Medicine, Seoul, Republic of Korea; 11 Public Health and Medical Service, Seoul National University Hospital, Seoul, Republic of Korea

**Keywords:** Acquired immunodeficiency syndrome, Human immunodeficiency virus, Health-related quality of life, Item response theory, Medical outcomes study HIV health survey

## Abstract

**Background:**

Precise assessment of health-related quality of life (HRQOL) with a reliable and valid measure is a prerequisite to the enhancement of HRQOL. This study examined the psychometric properties of the Korean version of the Medical Outcomes Study HIV Health Survey (K-MOS-HIV).

**Methods:**

The reliability and validity of the K-MOS-HIV were examined in a multicenter survey involving 201 outpatients with human immunodeficiency virus (HIV)/ acquired immunodeficiency syndrome (AIDS) from four teaching hospitals throughout Korea.

**Results:**

Ceiling effects were observed in six subscales scores, particularly, for the role functioning (71.1%), social functioning (63.2%), and pain (48.8%) scores. The Cronbach’s α for the physical health summary and mental health summary were 0.90 and 0.94, respectively, and it ranged from 0.78 to 0.95 for the subscales. The results of the exploratory structural equation modeling supported the two-factor structure of the K-MOS-HIV (physical health summary and mental health summary). An examination of the mean square statistics values from the Rasch analysis showed that the information-weighted fit and outlier-sensitive fit statistics were within the acceptable ranges of 0.6–1.4 except for two items in the mental health summary. The convergent validity of the K-MOS-HIV was supported by its significant positive correlations with the World Health Organization Quality of Life-HIV-BREF subscale scores. Its known-group validity was proven with its ability to detect significant differences in several K-MOS-HIV subscale scores among participants with different sociodemographic and clinical characteristics.

**Conclusions:**

The K-MOS-HIV health survey appears to be a reliable and valid measure of HRQOL.

## Background

As of 2015, 36.7 million persons are living with human immunodeficiency virus (HIV) worldwide [1]. With progress in treatment such as the introduction of highly active antiretroviral therapy (HAART), HIV has become a chronic illness with a significant decrease in HIV-related morbidity and mortality [2].

However, HIV/ acquired immunodeficiency syndrome (AIDS) still negatively affects the health-related quality of life (HRQOL) in people with HIV and AIDS (PLWHA) [3, 4]. HRQOL in PLWHA was significantly lower as compared to that of the general population [3] and those with other chronic diseases [5]. The stigma associated with HIV/AIDS is one worsening factor affecting HRQOL [6, 7]. Moreover, according to a prospective cohort study, HRQOL, particularly physical QOL, was a prognostic factor of survival among persons with HIV [8]. As such, attention to HRQOL-related issues and clinical efforts to enhance HRQOL in PLWHA are warranted. The precise assessment of HRQOL with a reliable and valid measure is a prerequisite to improve HRQOL. The World Health Organization Quality of Life-HIV-BREF (WHOQOL-HIV BREF) [9], Functional Assessment of HIV infection quality of life instrument (FAHI) [10], and the Medical Outcomes Study HIV Health Survey (MOS-HIV) [11] are some of the HIV/AIDS-specific tools that are used widely to assess the HRQOL of PLWHA. In particular, the FAHI and MOS-HIV are considered two of the most appropriate HIV-targeted HRQOL measures [12].

Among them, the application of the MOS-HIV to assess HRQOL is useful in two ways. Firstly, it allows between-group comparisons (e.g., populations with other diseases and healthy populations) as the MOS-HIV was developed from generic MOS instruments. It also permits within-group and cross-cultural comparisons as it has been applied to PLWHA worldwide, enabling the use of a vast amount of accumulated data for such comparisons [13]. The feasibility of the use of the MOS-HIV has been proven [14, 15], and it has been validated in several languages such as Chinese [16, 17], French [18], German [18], Greek [19], Italian [20, 21], Spanish [14], Thai [22] and Luganda [23].

The number of PLWHA in South Korea is increasing consistently, with an average of 1000 new cases reported each year since 2011. As of 2016, the number of PLWHA was 11,439 [24]. However, research regarding HRQOL in Korean PLWHA is limited. The strong stigma associated with the disease is prominent in Korea, as is the lack of a standardized and valid HRQOL tool. These seem to be some of the reasons behind this paucity of HRQOL research on the Korean population. Until now, only the WHOQOL-HIV BREF [25] has been translated and validated in Korean, and there is no validated Korean version of the MOS-HIV health survey. Thus, the purpose of the present study was to examine the psychometric properties of the Korean version of the MOS-HIV health survey in a sample of PLWHA in Korea.

## Methods

### Participants and procedures

Participants consisted of 201 outpatients with HIV/AIDS recruited via consecutive sampling. This cross-sectional, multi-center survey implemented between December 2016 and June 2017 involved four university hospitals in South Korea (two located in Seoul, the capital city, and the other two in the cities of Seongnam and Busan). On the provision of informed consent, the participants were asked to complete the survey, and they could complete it either on site or take it home and return it by mail. A gift certificate (equivalent to $9 U.S. dollars) was provided as a reward to all participants. This study was approved by the respective Institutional Review Boards of the participating institutions.

### Measures

The MOS- HIV, developed by Albert Wu and colleagues, is a self-report measure to assess HRQOL for patients with HIV/AIDS [26]. It has 35 items evaluating the following 11 dimensions of HRQOL: general health perception; pain; physical functioning; role functioning; social functioning; energy/fatigue; mental health; health distress; cognitive functioning; quality of life; and health transition. It also provides two summary scores of physical health and mental health, calculated from the scores of 10 subscales, excluding the health transition subscale [27]. While the scores on the physical functioning, pain, and role functioning scores contribute more strongly to the physical health summary score, those on mental health, health distress, quality of life, and cognitive functioning contribute more strongly to the mental health summary score. Scores on general health perception, energy/fatigue, and social functioning contribute to both factors [27]. Respondents rate items on a three-, five- and six-point Likert scale, and on a dichotomous yes/no scale, with reference to the last 4 weeks. In accordance with the MOS-HIV scoring instructions [28], the 11 items are recoded, and subscale scores as well as the two summary scores are computed. The MOS-HIV scores range from 0 to 100, with higher scores indicating better HRQOL.

The Korean version of the MOS-HIV (K-MOS-HIV) underwent a forward-backward translation procedure according to the European Organization for the Research and Treatment of Cancer translation guidelines [29]. Forward translation was performed by two independent clinicians, and the two versions of the Korean translation were integrated into a single version that underwent back-translation by an English speaker who has a good command of the Korean language and was blinded to the original version of the MOS-HIV. The developer of the MOS-HIV examined the back-translated version of the MOS-HIV and confirmed structural and semantic equivalence.

Moreover, the Korean version of the World Health Organization Quality of Life-HIV-BREF (K-WHOQOL-HIV BREF) [9, 25] was used as a criterion measure to determine the convergent validity of the K-MOS-HIV. The WHOQOL-HIV BREF has 31 items with 29 items divided into six domains of HRQOL in PLWHA (i.e., physical QOL, psychological QOL, level of independence, social relationships, environment, and spiritual QOL) and two items assessing an individual’s overall perception of QOL and health, respectively. Items are rated on a five-point Liker scale, and six domain scores are computed in accordance with the scoring guidelines [30]. Domain scores range from 4 to 20, with higher scores denoting higher QOL. The Cronbach’s α of the K-WHOQOL-HIV BREF is 0.94 [25].

Questions regarding sociodemographic data (i.e., age, sex, education, marital status, religion, employment, income, and insurance status) were included in a self-report survey. Clinical information (i.e., the Center for Disease Control and Prevention stage, years since diagnosis, CD4 cell counts, HIV RNA log, active antiretroviral therapy (ART) duration, psychological comorbidity, and medical comorbidity) were retrieved from medical records. A question regarding mode of transmission was also included in the survey, and participants were asked to choose among the options of sex with a person of the opposite sex, sex with a person of the same sex, and others.

### Statistical analyses

To determine whether the scale had adequate range variability, the floor and ceiling effects of the K-MOS-HIV were evaluated by examining percentages of scores at the extremes of the scaling range [19, 31].

The construct validity of the K-MOS-HIV was determined by examining its factorial and convergent validity. To determine its factorial validity, a confirmatory factor analysis (CFA) and exploratory structural equation modeling (ESEM) for a two-factor structure (physical health summary and mental health summary) were performed. In clinical research, many clinical symptoms or associated characteristics are likely to be associated with each other [32]. Therefore, the CFA, which requires the restriction of zero cross-loadings, might be restrictive, resulting in biased estimates [32, 33]. The ESEM, which is an integration of the best aspects of CFA, structural equation modeling, and exploratory factor analysis, does not require a strict restriction of zero cross-loading that causes over-estimated factor correlations and subsequent distorted structural relations [34]. Therefore, the model-fit of the CFA and ESEM for a two-factor structure was evaluated. The following criteria were applied to evaluate the goodness-of-fit indices of the factor models: standardized root mean square residual (SRMR) ≤ .08, comparative fit index (CFI) ≥ .95, Tucker-Lewis Index (TLI) ≥ .95, root mean square error of approximation (RMSEA) ≤ .08, and 90% confidence interval (CI) of RMSEA [35]. The factorial validity of the K-MOS-HIV was also examined by testing the partial credit model (PCM) in the polytomous model of a Rasch analysis [36]. The PCM is considered to be appropriate for analyzing responses in attitude or personality scales in which respondents rate their beliefs, or respond to statements on a multi-point scale [37]. The Rasch analysis provides two mean square (MNSQ) statistics, the outlier-sensitive fit statistic (outfit) and information-weighted fit statistic (infit) MNSQ. The outfit MNSQ is sensitive to outlier observations in the data, whereas the infit MNSQ is sensitive to unexpected inlying patterns among non-outlier observations. Therefore, the infit and outfit statistics were used to determine whether the item responses fit the expectations of the PCM. Both item MNSQ values of about 1.0 are considered ideal, and the values between 0.6 to 1.4 are deemed acceptable [38]. Higher MNSQ values (e.g., > 1.4) indicate a lack of construct homogeneity with other items in a scale, while lower values (e.g., < 0.6) indicate potential redundancy of the item with the rest of the scale [38, 39].

The convergent validity of the K-MOS-HIV was examined by examining its correlations with the K-WHOQOL-HIV BREF subscales scores. To examine known-group validity, *t*-tests or analyses of variance (ANOVA) were performed to examine whether the MOS-HIV scores differed among participants with varying sociodemographic and clinical characteristics that were known to be related to the HRQOL of PLWHA in a prior review [40]. Statistical analyses were performed using the IBM SPSS statistical package (version 23.0), M*plus* software (version 7.4), and jMetrik (version 4.0.5).

## Results

### Sociodemographic and clinical characteristics of participants

The participants’ characteristics in sociodemographic and clinical characteristics have been shown in Table 1. There were missing values (3 to 39) in all variables except age. The results of the Little’s missing completely at random (MCAR) [41] test indicated that the missing values were MCAR, and thus they did not affect the analyses (*X*^2^_(785)_ = 50.42, *p* = 1.00).

**Table 1:** Participant characteristics (*N* = 201)

	Total	Male (*n* = 179)	Female (*n* = 22)		
Variables	*N* (%) or *M* (*SD*)	*N* (%) or *M* (*SD*)	*N* (%) or *M* (*SD*)	*X*^2^/*t*	*p*
*Sociodemographic variables*					
Age (*n* = 201)	48.49 (13.33)	47.35 (13.13)	57.73 (11.41)	−3.54	< .001
≤ 40	54 (26.9)	52 (29.1)	2 (9.1)	24.10	< .001
41–60	107 (53.2)	100 (55.9)	7 (31.8)		
> 60	40 (19.9)	27 (15.1)	13 (59.1)		
Education (*n* = 197)					
Less than high school	45 (22.8)	30 (17.1)	15 (68.2)	30.09	< .001
High school	63 (32.0)	58 (33.1)	5 (22.7)		
College or above	89 (45.2)	87 (49.7)	2 (9.1)		
Marital status (*n* = 191)					
Not married (single/divorced/widowed)	142 (74.3)	127 (75.1)	15 (68.2)	.50	.48
Married	49 (25.7)	42 (24.9)	7 (31.8)		
Religion (*n* = 194)					
Yes	106 (54.6)	91 (52.9)	15 (68.2)	1.84	.18
No	88 (45.4)	81 (47.1)	7 (31.8)		
Employment (*n* = 190)					
Employed	95 (50.0)	89 (52.7)	6 (28.6)	4.34	< .05
Unemployed	95 (50.0)	80 (47.3)	15 (71.4)		
Income (*n* = 190)					
≤ 1 million Korean won (KRW)	65 (34.2)	52 (31.0)	13 (59.1)	7.14	< .05
1–3 million KRW^a^	70 (36.8)	64 (38.1)	6 (27.3)		
3 million KRW	55 (28.9)	52 (31.0)	3 (13.6)		
Insurance (*n* = 176)					
National health insurance	117 (66.5)	105 (67.3)	12 (60.0)	.43	.52
Medical aid	59 (33.5)	51 (32.7)	8 (40.0)		
*Clinical variables*					
CDC Stage (*n* = 195)					
A (Asymptomatic)	67 (34.4)	57 (32.8)	10 (47.6)	2.07	.36
B (Symptomatic)	68 (34.9)	63 (36.2)	5 (23.8)		
C (AIDS)	60 (30.8)	54 (31.0)	6 (28.6)		
Years since diagnosis (*n* = 162)	8.26 (5.97)	8.05 (6.06)	10.09 (4.89)	−1.34	.18
ART duration (year) (*n* = 187)	7.09 (5.37)	6.89 (5.54)	8.91 (3.10)	−2.44	.02
CD4 cell counts (*n* = 198)	649.09 (306.96)	642.24 (314.01)	703.91 (242.24)	−.89	.38
< 200 cells/ml	9 (4.5)	9 (5.1)	0 (.0)	1.18	.28
≥ 200 cells/ml	189 (95.5)	167 (94.9)	22 (100.0)		
HIV RNA log (*n* = 198)					
Not detected	139 (70.2)	123 (69.9)	16 (72.7)	.24	.97
< 40	43 (21.7)	39 (22.2)	4 (18.2)		
40–2000	9 (4.5)	8 (4.5)	1 (4.5)		
> 2000	7 (3.5)	6 (3.4)	1 (4.5)		
Psychological comorbidity (*n* = 198)					
Yes	30 (15.2)	25 (14.2)	5 (22.7)	1.11	.29
No	168 (84.8)	151 (85.8)	17 (77.3)		
Medical comorbidity (*n* = 198)					
Yes	182 (91.9)	164 (93.2)	18 (81.8)	3.40	.07
No	16 (8.1)	12 (6.8)	4 (18.2)		
Mode of transmission (*n* = 196)					
Sex with a person of the opposite sex	60 (30.6)	44 (25.3)	16 (72.7)	23.77	< .001
Sex with a person of the same sex	106 (54.1)	104 (59.8)	2 (9.1)		
Other	30 (15.3)	26 (14.9)	4 (18.2)		

^a^1–3 million KRW = equivalent to U.S. dollar $872 – $2616

The mean age of participants was 48.49 (*SD* = 13.33) years. Majority of participants were male (89.1%), not married (74.3%), educated up to college or higher (45.2%), professed a religion (54.6%), and on the national health insurance plan (66.5%). In terms of employment and income status, in general, participants were evenly distributed across categories.

As for clinical variables, approximately one-third of the participants were distributed at each of the following three stages of the Center for Disease Control and Prevention (CDC) classification system: A (asymptomatic), B (symptomatic), and C (AIDS). The mean for the number of years since diagnosis was 8.26 (*SD* = 5.97) years, and the mean duration of active antiretroviral treatment (ART) was 7.09 (*SD* = 5.37) years. The overall HIV-related condition of the participants was good, with 95.5% having CD4 cell counts of ≥200 cells/ml, and 70.2% having HIV RNA log (viral load) not detected. However, the rate of medical comorbidity was high (91.9%), while that of psychological comorbidity was 15.2%. Among the 30 participants who had psychological comorbidity, 24 had major depressive disorder, 1 had bipolar I disorder, 3 had insomnia, 1 had snoring/sleep apnea, and 1 had cognitive dysfunction problems. Additionally, majority of the participants were infected via sexual transmission, with 30.6% by heterosexual transmission and 54.1% by same sex transmission.

The examination of the sociodemographic and clinical characteristics by sex indicated significant differences between male and female participants in terms of age, education, employment, income, ART duration, and mode of transmission. Specifically, male participants were younger, had a higher percentage of college or above education, and were employed. With reference to income, the overall percentage of an income less than 1 million Korean Won (equivalent to U.S. dollar $872) was higher among female participants. The mean for the number of years of ART duration was shorter in male participants. For mode of transmission, the percentage of heterosexual transmission was higher among female, and that of the same sex transmission was higher among male participants.

### Scale properties and internal consistency of the K-MOS-HIV

The descriptive statistics for the subscale scores and the data on information for floor and ceiling effects have been presented in Table 2. The mean scores of the K-MOS-HIV subscale scores ranged from 53.42 (general health perception) to 85.87 (social functioning). Further, while a moderate flooring effect (> 15%) was observed for the role functioning scores (20.9%), substantial ceiling effects were observed in six subscales scores, especially for the role functioning (71.1%), social functioning (63.2%), and pain (48.8%) scores.

**Table 2:** Scale properties and internal consistency of the K-MOS-HIV and correlations between K-MOS-HIV and K-WHOQOL-HIV BREF^†^

	K-MOS-HIV												
K-WHOQOL-HIV BREF	GHP	P	PF	RF	SF	EF	MH	HD	CF	QL	HT	PHS	MHS
Overall QOL	.63^**^	.34^**^	.32^**^	.46^**^	.36^**^	.62^**^	.65^**^	.58^**^	.41^**^	.73^**^	.38^**^	.48^**^	.72^**^
General health	.75^**^	.46^**^	.37^**^	.53^**^	.44^**^	.65^**^	.61^**^	.68^**^	.45^**^	.62^**^	.32^**^	.59^**^	.73^**^
Physical QOL	.73^**^	.58^**^	.49^**^	.54^**^	.51^**^	.74^**^	.72^**^	.75^**^	.54^**^	.65^**^	.31^**^	.67^**^	.81^**^
Psychological QOL	.66^**^	.44^**^	.35^**^	.47^**^	.39^**^	.72^**^	.71^**^	.59^**^	.51^**^	.69^**^	.35^**^	.53^**^	.76^**^
Level of independence	.68^**^	.46^**^	.57^**^	.65^**^	.47^**^	.68^**^	.62^**^	.58^**^	.48^**^	.55^**^	.41^**^	.71^**^	.68^**^
Social relationships	.53^**^	.41^**^	.40^**^	.44^**^	.44^**^	.59^**^	.59^**^	.50^**^	.41^**^	.55^**^	.24^**^	.53^**^	.63^**^
Environment	.50^**^	.44^**^	.40^**^	.41^**^	.36^**^	.59^**^	.55^**^	.48^**^	.36^**^	.55^**^	.23^**^	.51^**^	.59^**^
Spirituality	.53^**^	.32^**^	.22^**^	.37^**^	.36^**^	.63^**^	.63^**^	.64^**^	.52^**^	.57^**^	.27^**^	.39^**^	.71^**^
												Range	
*M*	53.42	83.80	76.75	75.63	85.87	60.39	65.16	70.75	79.40	56.72	55.10	24.08 - 64.77	14.13 - 65.88
*SD*	24.11	20.79	25.11	40.87	22.44	20.50	21.19	27.79	20.67	22.04	19.74		
Cronbach’s α	0.88	0.82	0.86	0.90	–	0.78	0.78	0.95	0.89	–	–	0.90	0.94
No. of Items	5	2	6	2	1	4	5	4	4	1	1		
% Floor	3.0	2.5	0.5	20.9	1.5	1.0	0.5	1.5	0.5	5.0	3.0		
% Ceiling	2.5	48.8	33.8	71.1	63.2	3.0	3.5	24.4	25.4	5.5	8.5		

*GHP* General Health Perception, *P* Pain, *PF* Physical Functioning, *RF* Role Functioning, *SF* Social Functioning, *MH* Mental Health, *EF* Energy/Fatigue, *HD* Health Distress, *CF* Cognitive Functioning, *QL* Quality of Life, *HT* Health Transition, *PHS* Physical Health Summary Score, *MHS* Mental Health Summary Score; **p < .01

† Sample size varies from 193 to 201 due to missing data

The Cronbach’s α of the K-MOS-HIV scales as a measure of internal consistency, was satisfactory overall, with values ranging from 0.78 to 0.95 across subscales. Further, those for the physical health summary and the mental health summary were 0.90 and 0.94, respectively.

### Construct validity of the K-MOS-HIV: Factorial and convergent validity

**Fig. 1:**
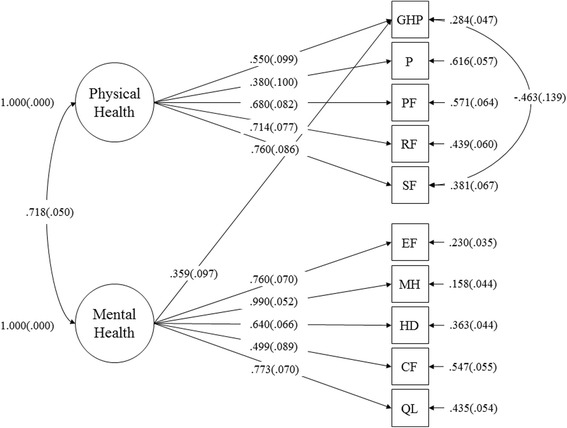
Standardized estimates in the exploratory structural equation modeling of the K-MOS-HIV (*N* = 201)

The results of the CFA examining the factorial validity of the K-MOS-HIV indicated that the proposed two-factor structures (physical health summary and the mental health summary) represented by the subscales of the MOS-HIV showed unsatisfactory goodness of fit indices (*Χ*^2^_(32)_ = 501.9, *p* < .001, RMSEA = .270 [.250–.291], CFI = .608, TLI = .449, and SRMR = 1.367). On the other hand, the results of the ESEM (Fig. 1), which specified a residual correlation between general health perception and social functioning (modification index = 13.65, standardized expected parameter change = −.34), demonstrated an adequate fit to the data (*Χ*^2^_(25)_ = 56.2, *p* < .001, RMSEA = .079 [.051–.106], CFI = .974, TLI = .953, and SRMR = .027). While social functioning, role functioning, and physical functioning loaded on the physical health summary, mental health, quality of life, energy/fatigue, health distress, and cognitive functioning loaded on the mental health summary (Table 3). General health perception and pain loaded more on the physical health summary, but it also loaded to a lesser degree on the mental health summary.

**Table 3:** Factor loading matrix of the ESEM model (*N* = 201)

Scales	PHS	MHS
General health perception	.55***	.36**
Pain	.38***	.29**
Physical functioning	.68***	−.04
Role functioning	.71***	−.05
Social functioning	.76***	−.04
Energy/Fatigue	.15*	.76***
Mental health	−.10	.99***
Health distress	.20**	.64***
Cognitive functioning	.22*	.50***
Quality of life	.03	.77***

Factor loading with magnitudes > .35 have been shown in bold; *p < .05, **p < .01, ***p < .001

The convergent validity of the K-MOS-HIV was demonstrated by its significant positive correlations with the K-WHOQOL-HIV BREF subscale scores. All correlations were significant (Table 2) and the correlation coefficients ranged from .22 to .81. Both the physical and psychological domain scores of the K-WHOQOL-HIV BREF were significantly correlated with the respective physical health summary (*r* = .67, *p* < .01) and mental health summary scores (*r* = .76, *p* < .01). However, the correlation of the K-WHOQOL-HIV BREF physical domain score with the mental health summary of the K-MOS-HIV (*r* = .81, *p* < .01) was slightly stronger as compared to the physical health summary of the K-MOS-HIV.

### Results of Rasch analysis

The results of the Rasch analysis have been shown in Table 4. The data from participants with missing responses on one or more items were excluded from the Rasch analysis (*n* = 16). To evaluate the content homogeneity or unidimensionality of the items within each identified factor, the Rasch measurement model was used. Following the results of the ESEM supported the two-factor structure of the K-MOS-HIV, the Rasch analysis was performed for these two factors.

**Table 4:** Infit and outfit MNSQ statistics, item difficulties, and step parameters for each item (*N* = 185)

Item	Infit	Outfit	Diff.	Step 1	Step 2	Step 3	Step 4	Step 5
PHS								
GHP 1	1.07	1.09	1.68	−3.41	−1.60	1.40	3.61	–
P2	1.37	1.37	−0.87	−1.48	−1.15	0.37	0.63	1.63
P3	0.97	1.04	−1.19	−1.44	−0.68	**1.14**	**0.98**	–
PF 4a	1.01	1.04	0.54	−1.12	1.12	–	–	–
PF 4b	0.84	0.76	− 0.24	− 0.74	0.74	–	–	–
PF 4c	0.96	1.18	−0.09	− 0.96	0.96	–	–	–
PF 4d	0.91	0.70	−0.47	− 0.79	0.79	–	–	–
PF 4e	1.12	0.80	− 0.93	**0.20**	**− 0.20**	–	–	–
PF 4f	1.30	0.83	−1.02	**1.45**	**− 1.45**	–	–	–
RF 5	0.69	**0.50**	−0.41	**–**	**–**	**–**	**–**	**–**
RF 6	0.73	**0.54**	−0.23	–	**–**	**–**	–	–
SF 7	0.95	0.99	−0.81	**0.25**	**−1.32**	**−0.51**	**0.93**	**0.65**
GHP 11a	0.96	0.93	1.06	−2.21	**−0.17**	**−0.23**	2.61	
GHP 11b	1.19	1.20	1.01	−2.22	**−0.43**	**− 0.66**	3.31	
GHP 11c	1.05	1.00	1.36	−2.35	**−0.40**	**−0.59**	3.34	
GHP 11d	1.16	1.27	0.61	−1.82	**−0.06**	**− 0.37**	2.25	
MHS								
MH 8a	0.75	0.66	−0.45	−1.15	**− 0.24**	**−0.34**	0.61	1.12
MH 8b	**1.80**	**2.27**	0.74	−1.26	−0.79	**0.48**	**−0.14**	1.71
MH 8c	0.74	0.75	−0.16	−0.96	− 0.78	−0.57	1.07	1.25
MH 8d	**1.47**	**1.54**	0.82	−1.14	−0.81	0.15	0.62	1.18
MH 8e	1.25	1.07	−0.36	− 0.98	−0.59	− 0.05	0.60	1.02
EF 9a	1.13	1.13	0.76	−2.16	−1.16	0.45	1.17	1.69
EF 9b	0.82	0.84	−0.25	**−0.77**	**−0.96**	− 0.64	0.72	1.65
EF 9c	0.90	0.92	0.16	−1.60	−1.45	−0.18	1.02	2.21
EF 9d	1.30	1.28	0.59	−1.38	−1.14	0.17	0.94	1.41
HD 9e	0.73	0.73	0.01	**−0.36**	**−0.72**	**−0.81**	0.87	1.02
HD 9f	0.70	0.63	−0.02	**− 0.41**	**−0.70**	**− 0.48**	0.72	0.88
HD 9 g	0.65	0.61	−0.08	**−0.44**	**− 0.33**	**−0.69**	**0.93**	**0.53**
HD 9 h	0.91	0.88	0.01	−0.76	− 0.57	−0.31	0.52	1.12
CF 10a	0.79	1.33	−0.28	**−0.10**	**− 0.73**	**−0.49**	0.55	0.77
CF 10b	1.22	1.24	−0.55	**−0.52**	**−1.00**	**− 0.76**	0.91	1.37
CF 10c	0.86	0.78	−0.69	−1.20	**−0.19**	**− 0.79**	0.85	1.34
CF 10d	0.93	0.84	−0.93	**−0.62**	**−1.41**	− 0.25	1.11	1.17
QL 12	0.82	0.82	0.67	**−1.50**	**−2.17**	0.68	3.00	–

*GHP* General Health Perception, *P* Pain, *PF* Physical Functioning, *RF* Role Functioning, *SF* Social Functioning, *MH* Mental Health, *EF* Energy/Fatigue, *HD* Health Distress, *CF* Cognitive Functioning, *QL* Quality of Life, *PHS* Physical Health Summary Score, *MHS* Mental Health Summary Score

Infit and outfit MNSQ statistics higher than 1.4 or lower than 0.6 as well as disordered step parameters are shown in bold

On the physical health summary, all the items had a good fit, with the infit MNSQ ranging from 0.69 to 1.37 and outfit MNSQ ranging from 0.50 to 1.37. On the mental health summary, two items had both infit and outfit statistics greater than 1.4: the mental health 8b had an infit of 1.80 and outfit of 2.27; the mental health 8d had an infit of 1.47 and outfit of 1.54. All other items on the mental health summary demonstrated good fit, with the infit MNSQ ranging from 0.65 to 1.30 and outfit MNSQ ranging from 0.61 to 1.33.

The results regarding item difficulties and the step parameters for the 34 items indicated that not all items in all the scales maintain the given step orders. In majority of the items, only two-step values were not ordered. For instance, the four step parameters of the item Pain 3 were − 1.44, − 0.68, 1.14, and 0.98, showing that only the third (1.14) and fourth (0.98) steps were dislocated. However, six items showed that at least three step estimates were disordered (e.g., − 0.36, − 0.72, − 0.81, 0.87, and 1.02 for the item Health Distress 9e).

### Known-group validity

The results of the *t*-tests or ANOVAs conducted to examine the ability of the K-MOS-HIV to detect potential differences in various dimensions of HRQOL among participants with different sociodemographic and clinical characteristics have been summarized in Table 5. In terms of sociodemographic variables, a number of subscale scores showed significant differences among groups formed by age, marital status, employment, monthly income and insurance status.

**Table 5:** Known-group validity of the K-MOS-HIV^†^

	Scale^a, b^											PHS^a, b^	MHS^a, b^
	GHP	P	PF	RF	SF	EF	MH	HD	CF	QL	HT		
*Sociodemographic variables*													
Age^a^			***		*							**	
Sex													
Education													
Marital status^b^					**	*	*						*
Religion													
Employment^b^	**		***	***	**	**	*	**	***			***	**
Income^c^	***	**	***	***	*	***	**	**	**	*		***	**
Insurance^b^	***		***	**	**	*	*	*	*			***	*
*Clinical variables*													
CDC stage													
Years since diagnosis^b^											**		
ART duration^d^					*						**		
CD4 cell counts^b^	**		*	*	*	*						**	
HIV RNA log^e^					*								
Psychological Comorbidity^b^						**	**	*	**	*			**
Medical Comorbidity^b^			**	***	*							***	
Mode of transmission													

*GHP* General Health Perception, *P* Pain, *PF* Physical Functioning, *RF* Role Functioning, *SF* Social Functioning, *MH* Mental Health, *EF* Energy/Fatigue, *HD* Health Distress, *CF* Cognitive Functioning, *QL* Quality of Life, *HT* Health Transition; *p < .05, **p < .01, ***p < .001

†Sample size varies from 162 to 201 due to missing data;

^a^The post hoc (Bonferroni) tests indicated that PF (1 > 2 = 3) and PHS (1 > 2). 1 = (≤ 40), 2 = (41–60), 3 = (> 60)

^b^All results indicate that 1 > 2; 1 = married, 2 = not married; 1 = employed, 2 = unemployed; 1 = National health insurance, 2 = medical aid; 1 = (Years since diagnosis ≤ mean), 2 = (Years since diagnosis > mean); 1 = (CD4 ≥ 200/mm^3^), 2 = (CD4 < 200/mm^3^); 1 = Comorbidity no, 2 = Comorbidity yes

^c^All post hoc Bonferroni tests indicated that 1 < 2 = 3, with the exceptions of P (1 < 3) and SF (1 < 2). 1 = (≤ 1 million KRW), 2 = (1–3 million KRW), 3 = (> 3 million KRW)

^d^SF (1 < 2) and HT (1 > 2). 1 = (ART duration ≤ mean), 2 = (ART duration > mean)

^e^The post hoc (Bonferroni) tests indicated that 1 > 2 = 3 = 4; 1 = (Not detected), 2 = (< 40), 3 = (40–2000), 4 = (> 2000)

Age was associated with the physical functioning and social functioning subscales, *F*_(2, 198)_ = 9.67 and 3.74, and the physical health summary, *F*_(2, 198)_ = 5.01. However, for the social functioning subscale, the *post-hoc* analysis revealed that the between-group difference was not significant. For marital status, the subscale scores on social functioning, energy/fatigue, and Mental Health as well as the mental health summary were higher among married participants than non-married counterparts, *t*_(189)_ = 2.24, 2.60, 2.26, and 2.00, respectively. Employed participants showed higher scores, as compared to their unemployed counterparts, on eight dimensions of the K-MOS-HIV (general health perception, physical functioning, role functioning, social functioning, energy/fatigue, mental health, health distress, and cognitive functioning) as well as on the two summary scores of physical health and mental health, *t*_(188)_ = 2.92, 5.30, 4.11, 2.65, 2.80, 2.40, 2.91, 3.33, 4.29, and 3.09, respectively. Participants with higher income showed higher scores than did those with lower income on all 10 MOS-HIV subscales and on the physical health summary and mental health summary, *F*_(2, 187)_ = 10.00, 5.60, 23.80, 13.17, 4.50, 7.31, 4.78, 5.60, 5.27, 3.24, 19.68, and 6.63, respectively. Furthermore, the participants on the national health insurance plan showed a better QOL than those with medical aid, showing similar patterns as those observed in employment, *t*_(174)_ = 3.75, 5.06, 3.50, 3.13, 2.31, 2.17, 2.36, 2.18, 4.87; and 2.50, respectively.

With reference to the clinical variables, participants with a CD4 cell count of above 200 cells/ml showed higher scores on five subscales (general health perception, physical functioning, role functioning, social functioning, and energy/fatigue) and on the physical health summary, *t*_(196)_ = 2.76, 2.30, 2.27, 2.30, 1.99, and 2.91, respectively. The social functioning scores differed according to viral load status (*F*_(3, 194)_ = 3.07) and ART duration, *t*_(185)_ = − 2.52. Additionally, participants with a shorter ART duration and years since diagnosis showed higher health transition scores, *t*_(185)_ = 3.02 and *t*_(160)_ = 2.85, respectively. Participants with a comorbid psychiatric disorder showed lower scores on five subscales (energy/fatigue, mental health, health distress, cognitive functioning, and quality of life) and on the mental health summary, *t*_(196)_ = − 2.79, − 2.85, − 2.44, − 3.10, − 2.08, and *−* 3.12, respectively. Moreover, participants with a medical comorbidity showed lower scores on three subscales (physical functioning, role functioning, and social functioning), and on the physical health summary, *t*_(196)_ = − 2.04, − 4.63, − 2.19, and − 4.07, respectively.

## Discussion

This study examined the reliability and validity of the K-MOS-HIV in a multicenter study involving 201 outpatients with HIV/AIDS in Korea.

Firstly, ceiling effects were observed in six subscales, particularly in role functioning (71.1%), social functioning (63.2%), and pain (48.8%), while a floor effect was observed in role functioning (20.9%). The observed pattern of these effects (e.g., ceiling effects for role functioning, social functioning, and pain) is line with the results obtained from validation studies of the MOS-HIV in several languages [16, 19, 42]. For example, ceiling effects in role functioning (79%), social functioning (76%) and floor effect in role functioning (14.5%) were reported in the Thai version of the MOS-HIV [22]. The ceiling effects observed in the present study might be related to the relatively good level of HIV-related condition of the participants, as evidenced by clinical characteristics such as 95.5% of the participants having CD4 cell counts of ≥200 cells/ml and 92% having an HIV RNA log value less than 40. In fact, majority of the participants in previous studies that reported similar patterns of ceiling or floor effects had CD4 cell counts of > 200 cells/ml [19, 42]. On the other hand, floor effects in social functioning (21.2%) and role functioning (72.6%) were observed in the Italian version of the MOS-HIV [20], with only 7.6% of participants having CD4 cell counts of > 200 cells/ml [20].

Regarding the factorial validity of the K-MOS-HIV, the results of the ESEM supported its previously proposed two-factor structure (physical health summary and mental health summary) [27]. However, the loading patterns of subscales of the physical health summary and mental health summary were somewhat different. For instance, in Revicki et al.’s study [27], pain, role functioning, and physical functioning loaded on the physical health summary, while mental health, health distress, quality of life and cognitive functioning loaded on the mental health summary, and general health perception, social functioning and energy/fatigue loaded on both factors. However, in our study, social functioning loaded on the physical health summary, and energy/fatigue loaded only on the mental health summary. Additionally, general health perception and pain loaded on both factors. In Lau et al.’ study [16], energy/fatigue loaded on the mental health summary (factor loading .71) and pain loaded on both the physical health summary (loading value of .54) and mental health summary (.26). In the Thai version of the MOS-HIV [22], while pain loaded mainly on the physical health summary (.62), it also loaded on the mental health summary (.35). The multidimensional nature of pain [43, 44], as well as the association of pain with mental health problems previously observed in HIV-infected adults, [45, 46] might explain this variation. For instance, the level of anxiety and depression was higher in patients with HIV reporting pain than in those who did not [45]. Similarly, HIV+ persons with diagnosed mood/anxiety and substance use disorders reported significantly higher levels of pain than did HIV+ persons without these comorbid conditions [47]. The loading of energy/fatigue on the mental health summary may be explained by a significant association between fatigue and depression previously observed in PLWHA [48, 49].

Furthermore, an examination of the MNSQ values from the Rasch analysis showed that the infit and outfit statistics were within acceptable ranges of 0.6–1.4, except for two items in the mental health summary, indicating the unidimensionality of the items within each factor.

However, in terms of the step parameter, step disorders were observed in 19 out of 34 items. This might be a result of the small sample size, and in fact, a sample size larger than 250 was suggested to ensure an adequate and stable estimation of parameters for Rasch analysis [50, 51]. Furthermore, this might be related to the relatively good HIV-related condition of the study participants, as evidenced by their CD4 cell counts and viral load status, as well as their scores on K-MOS-HIV. Eleven out of the 19 items with step disorders were those which showed ceiling effects. The patterns of step disorder might be related to the relatively good functioning level of the participants. A small number of participants selected the lower level response category. Re-examination of the K-MOS-HIV with a sufficient number of participants with varying functioning levels is therefore necessary.

With regard to the known-group validity of the K-MOS-HIV, a number of subscale scores differed significantly across groups with varying sociodemographic (i.e., age, marital status, employment, monthly income and insurance) and clinical characteristics (i.e., years since diagnosis, ART duration, CD4 cell counts, HIV RNA log, psychological, and medical comorbidity). With regard to sociodemographic variables, younger participants showed higher physical functioning and physical health summary scores. This inverse association between age and physical health observed in the present study is in line with the findings of a previous review [40]. In a study with 1191 HIV-positive patients, participants aged over 45 years reported lower scores on the physical domain of the HRQOL than did those aged under 34 years [52]. Relatedly, a previous study found that nearly half of older people with HIV had at least one major medical comorbidity, and a greater burden of co-morbidity was associated with a lower physical HRQOL [53]. Similarly, the independent effects of age observed for physical functioning and general health perceptions, as well as significant interaction effects of HIV and older age on daily functioning, were observed in PLWHA [54]. Further, in the present study, married people showed higher social functioning, energy/fatigue, mental health and mental health summary scores, which might be understood from a previously observed link between social support and better physical health summary and mental health summary in PLWHA [40, 55].

The results also suggested that being employed was associated with an overall higher HRQOL in PLWHA, which was in line with previous findings [56–58]. In fact, employment status was the strongest predictor of the five MOS-HIV health survey subscales scores in PLWHA in Canada [57]. Similarly, Rueda and colleagues [56] found that the effect of employment on QOL was comparable to that of higher HIV-related symptoms. They also suggested that the association between employment and QOL is to be understood in both directions, that is, overall good health being a necessary prerequisite to get or maintain employment (i.e., “the selection process”), and unemployment status causing lower QOL (i.e., “the causation process”). Moreover, insurance and income showed a similar pattern, which is in line with previous findings. For instance, lower income was associated with worse physical and mental health [5], and increased suicide risk [59].

Among clinical variables, there were no significant differences in the MOS-HIV scores in terms of CDC stage. However, as prior studies suggest that the association of advanced disease with physical health [40] and all the domains of the MOS-HIV except health transition [60], the ability of the MOS-HIV to discriminate HRQOL status according to the disease stage is unclear. For instance, the Spanish version of the MOS-HIV did not discriminate well between disease stages [14]. On the other hand, in a study with the Greek version of the MOS-HIV, scores on the physical functioning and pain subscales, and the physical health summary were significantly lower in individuals with AIDS compared to those with asymptomatic HIV+ [19].

Moreover, the present results indicated that the physical health summary scores as well as those on the subscales that loaded on the physical health summary such as general health perception, physical functioning, role functioning, and social functioning were significantly lower in groups with low CD4 cell counts or the group with a medical comorbidity. Similarly, the mental health summary scores as well as those on the subscales comprising the mental health summary, such as energy/fatigue and mental health, were lower in the group with a psychological comorbidity, confirming the known-group validity of the MOS-HIV. Although previous findings regarding the association between CD4 cell counts and MOH-HIV scores are mixed, majority of these findings revealed a significant association of higher CD4 cell counts with higher HRQOL [19, 61]. For instance, as compared to those with CD4 cell counts over 200, the group with CD4 cell counts less than 200 scored lower on the general health perception, physical functioning, pain, and energy/fatigue subscales, and the physical health summary in the MOS-HIV validation study conducted in Taiwan [42]. Similarly, patients with CD4 cell counts less than 200 showed lower physical well-being and overall QOL than did those with CD4 cell counts over 200 [62]. Additionally, in the present study, social functioning scores differed according to viral load status, which is partially consistent with a previously observed negative association of physical and mental health summary scores with viral loads [63]. The inverse association of comorbidity burden with overall HRQOL, particularly with physical QOL, was in line with that observed in previous studies on PLWHA [53, 64]. Furthermore, psychiatric comorbidity was related to lower mental health summary scores in the present study, which is consistent with previous findings that observed the association of depression with diminished HRQOL [60, 65].

The current results should be considered with their limitations. First, the cross-sectional nature of this study precludes any causal interpretation regarding the associations of sociodemographic and clinical characteristics with several dimensions of the MOS-HIV observed in this study. Additionally, due to the consecutive sampling procedure employed, the possibility of sample bias should be considered, and in fact, the ceiling effects in a number of subscale scores observed in this study might be related to the relatively good level of overall HIV-related health condition of the participants. Those with a poorer condition were more likely to decline study participation. However, in terms of sex, age, and mode of transmission of HIV, the characteristics of our study participants are similar to those of people living with HIV/AIDS in South Korea [24]. To explain, 89.1% of our study participants were male, similar to 92.8% of males in people living with HIV/AIDS in 2016. In addition, in our study, 26.9% of the participants were aged below 40 years, 53.2% of them were aged between 41 and 60 years, and 19.9% of them were aged over 60 years, as compared to the percentages of 38.6, 48.1, and 13.3% reported among PLWHA in South Korea in 2016, respectively [24]. The percentages of heterosexual and same-sex transmission were 30.6 and 54.1%, respectively, as compared to 58.4 and 41.5%, respectively, which were reported among notified cases of HIV during 2007–2016 [24].

Moreover, for a HRQOL measure used in clinical practice, it is necessary to assess the sensitivity of the tool to detect changes in clinical status over time [66, 67]. However, the test-retest reliability and the sensitivity of the K-MOS-HIV to detect changes or responsiveness to symptom changes [68] was not tested in the present study, and they should be examined in a longitudinal and prospective study.

## Conclusion

Despite these limitations, the present results suggest that the K-MOS-HIV is a reliable and valid measure of HRQOL in PLWHA, which would allow related research as well as cross-cultural comparisons on HRQOL in this population.

## Abbreviations

AIDS: Acquired immunodeficiency syndrome
CF: Cognitive functioning
CFA: Confirmatory factor analysis
CFI: Comparative fit index
EF: Energy/fatigue
ESEM: Exploratory structural equation modeling
FAHI: Functional assessment of HIV infection quality of life instrument
GHP: General health perception
HAART: Highly active antiretroviral therapy
HD: Health distress
HIV: Human immunodeficiency virus;
HRQOL: Health-related quality of life;
HT: Health transition
INFIT: Information-weighted fit statistic
MH: Mental health
MHS: Mental health summary score
MNSQ: Mean square
MOS-HIV: Medical outcomes study HIV health survey
OUTFIT: Outlier-sensitive fit statistic
PCM: Partial credit model
PF: Physical functioning
PHS: Physical health summary score;
PLWHA: People with HIV and AIDS
QL: Quality of life
RF: Role functioning
RMSEA: Root mean square error of approximation
SF: social functioning;
SRMR: Root mean square residual
WHOQOL-HIV BREF: World health organization quality of life-HIV-BREF
